# Internal Bisphenol Analogue Exposure in an Elderly Chinese Population: Knowledge from Dietary Exposure

**DOI:** 10.3390/toxics13040259

**Published:** 2025-03-29

**Authors:** Xinjie Duan, Mengyuan Liang, Beibei Wei, Jie Gu, Qian Zhao, Guixiang Ji, Shengyang Jin, Huanhuan Chen

**Affiliations:** 1 Department of Endocrinology, The First Affiliated Hospital of Nanjing Medical University, No. 300, Guangzhou Road, Gulou District, Nanjing 210029, China; duanxinjie0307@163.com (X.D.); 18914712609@163.com (B.W.); 2 Nanjing Institute of Environmental Science, Ministry of Ecology and Environment, No. 8, Jiangwangmiao Street, Xuanwu District, Nanjing 210042, China; liangmengyuan@nies.org (M.L.); gujie@nies.org (J.G.); jgx@nies.org (G.J.); 3 Department of Endocrinology, Nanjing Lishui People’s Hospital, No. 86, Chongwen Road, Lishui District, Nanjing 211200, China; 4 Department of Endocrinology, Nanjing Liuhe District People’s Hospital, No. 28, Yanan Road, Liuhe District, Nanjing 211500, China; zq_19871011@163.com; 5 Development Area Branch of Lianyungang Municipal Bureau of Ecology and Environment, No. 601, Huaguoshan Road, Lianyungang Economic & Technological Development Area, Lianyungang 222069, China

**Keywords:** bisphenol analogues, internal exposure, influencing factors, exposure assessment

## Abstract

Due to its endocrine-disrupting effects and neurotoxicity, Bisphenol A (BPA) has been banned from some products and some countries; therefore, alternatives are increasingly being used. Studies have been performed to evaluate internal Bisphenol analogue (BP) exposure in children, adolescents and adults; however, little information on elderly age groups is available. In this study, a cohort of 161 senior residents aged 60–70 years, from a coastal residential district in Jiangsu Province of China, was selected, and blood samples were collected from these individuals to evaluate internal BP exposure. The serum concentrations of eleven BPs (BPA, BPB, BPC, BPE, BPF, BPS, BPZ, BPP, BPAF, BPAP and TBBPA) were quantitatively determined by HPLC-MS/MS. In parallel, demographic and dietary surveys were conducted, and the potential association between BP levels and dietary habits was analyzed. Noteworthily, the detection rate of 10 BPs in serum samples exceeded 78%. Of all the BPs, BPA displayed the highest level, followed by BPAF, BPB, and BPS. Interestingly, the levels of most types of BPs in males were higher than those in females, and individuals above 65 years of age exhibited significantly higher BPA levels. Dietary analysis indicated a significant correlation between meat consumption and BP levels, implying that this is an important source of BP exposure. The current study uncovers previously unknown aspects of BPs exposure, characterized by high internal BP levels in the elderly, and risk factors such as gender and meat consumption. This offers valuable insights for preventing region-specific BP exposure in the elderly.

## 1. Introduction

Environmental endocrine-disrupting chemicals (EDCs) are a class of exogenous substances that can cause endocrine toxicity, as well as developmental and reproductive toxicity [1]. Bisphenol A (BPA), a typical EDC, has been widely used in the production of polycarbonate (PC), epoxy resins, paints, food packaging and food containers since 1957 [2]. BPA has the ability to bind with both estrogen receptors, namely ERα and ERβ. While it exhibits a greater affinity for ERβ, its overall binding affinity to these receptors is considerably lower compared to that of 17β-estradiol [3]. Its ubiquitous presence in the environment has raised significant health concerns, due to its potential linkage to obesity, diabetes, eclampsia, hyperlipidemia, neoplasms, cardiovascular disease, chronic kidney disease, infertility, polycystic ovary syndrome, etc. [2,4,5,6,7,8]. Therefore, the European Union banned BPA-containing plastic baby bottles in 2011 [9]. Therefore, alternatives to BPA are increasingly being used, such as bisphenol F (BPF), bisphenol AF (BPAF) and bisphenol S (BPS). For example, BPS has found applications in the manufacturing of food packaging, baby bottles and thermal paper; BPF is predominantly utilized in epoxy resins, coatings and structural adhesives; and BPAF serves as a cross-linking agent in the electronics and fibre optics industries [10].

Since the usage of BPs has gradually increased, their exposure level urgently needs to be evaluated. Studies have revealed that humans are exposed to bisphenol analogues through a wide spectrum of media, such as air, food, drinking water and indoor dust [11]. For safety, the U.S. Environmental Protection Agency (U.S.EPA) set the maximum daily intake (MDI) of BPA at 0.05 mg/kg·bw/d in 1993, Health Canada set an MDI of BPA at 0.025 mg/kg·bw/d in 2008, and the European Food Safety Authority (EFSA) set an MDI of BPA at 0.05 mg/kg·bw/d in 2006, which was readjusted in 2015 to 0.004 mg/kg·bw/d [12]. Recently, an increasing number of studies have focused on measuring BP levels in humans [13,14,15,16]. For example, the median concentrations of BPA and BPS in urine samples from Guangdong, China, were 31.07 and 7.37 ng/mL, respectively [13]. Intriguingly, BPA and BPS levels were significantly higher in males than in females [13]. In a study examining the relationship between BPA and hyperlipidemia among the elderly population in Hubei, China, the median BPA concentration was determined to be 3.09 ng/mL, with a range extending from 0.08 to 8.14 ng/ml [14]. This study also identified several other Bisphenol analogues, with the following geometric mean (GM) concentrations: BPF (0.228 ng/mL), BPAF (0.018 ng/mL) and BPS (0.029 ng/mL) [14].

Nonetheless, the vast majority of studies on the issue of BP exposure are focused on BPA in the urine of children, adolescents and adults; information on its substitutes in blood (serum), as well as its risk factors, are still unknown. The purpose of this study was to evaluate the internal BP exposure in an elderly Chinese population; moreover, the underlying risk factors are also discussed in the present work.

## 2. Materials and Methods

### 2.1. Chemicals and Reagents

BPA, BPB, BPC, BPE, BPF, BPS, BPZ, BPP, BPAF and BPAP standards with purity ≥ 98% were purchased from J&K Scientific (Shanghai, China). Isotope internal standards, such as BPA-d6, BPS-d8, BPAF-d4, were purchased from Accustandard, Inc. (Market Street, New Haven, CT, USA).

HPLC-grade methanol, hexane and dichloromethane were purchased from Merck KGaA (Darmstadt, Germany). Deionized water was prepared by the Milli-Q^®^ Water Purification System (Billerica, MA, USA). Formic acid (HPLC-grade) was bought from Sigma-Aldrich (Saint Louis, MO, USA). A Hydrophilic–Lipophilic Balance (HLB) cartridge (500 mg, 6 mL) was bought from Waters Corporation (Milford, MA, USA).

### 2.2. Participant Sample Collection

This study employed a cross-sectional design to assess BP exposure and associated risk factors in an elderly population in a city on the eastern coast of Jiangsu Province. A total of 161 permanent residents aged 60–70 years were randomly selected from a residential community. Participants were included if they had resided in the community for at least 5 years. Blood samples (5 mL) were collected from the population; a self-designed questionnaire containing questions on age, gender and environmental and occupational risk factors was used to investigate the selected participants. Moreover, a dietary survey was carried out on the survey population. We distributed dietary questionnaires to the subjects of the survey, which included questions on daily dietary habits, types of food, food weight, etc., and required the respondents to carefully recall and fill in the information truthfully. Data were reviewed for completeness and plausibility. The study was reviewed and approved by the Ethics Committee of the First Affiliated Hospital of Nanjing Medical University (Jiangsu Provincial People’s Hospital) (Luncheon Review: No. 2023-SR-234). All study subjects signed an informed consent form.

### 2.3. Blood Sample Preparation

Samples were thawed at 4 °C, vortexed and mixed. To precipitate proteins, 400 μL of serum was pipetted into an Ep tube, and 800 μL of methanol solution containing 0.2% formic acid was added. Methanol was used to precipitate proteins from the serum samples, and formic acid (0.2%) was added to the methanol solution to improve the ionization of BPs during Liquid Chromatography–Tandem Mass Spectrometry (LC-MS/MS) analysis. The mixture was vortexed to precipitate the protein, transferred to a low-temperature centrifuge and centrifuged at 8000 rpm for 10 min. The supernatant was then transferred to a new cuvette, and 20 mL of purified water was added and mixed. The mixture was left to be purified.

A solid-phase extraction (SPE) column was pre-conditioned with sequential rinses of 5 mL of hexane, 5 mL of dichloromethane, 10 mL of methanol and 10 mL of laboratory-grade water to make it better integrated with the target. Following this preparation, the sample was then passed through the SPE column at a flow rate of 3 mL to 5 mL per minute, for adsorption of the targets. After this, the column was rinsed with 5 mL of 10% methanol aqueous solution to wash away non-specific/unwanted compounds. Then, the column was dried under a stream of nitrogen gas. The retained compounds were then eluted using 5 mL of methanol and 10 mL of dichloromethane. The collected eluate was concentrated under nitrogen to a final volume of less than 1 mL. The solvent was exchanged, and the sample was brought up to a fixed volume of 50 μL with methanol; subsequently, it was allowed to stand before proceeding to analysis. The serum samples were purified using solid-phase extraction (SPE), according to the method described by Maknun L et al. [17].

### 2.4. Instrumental Analysis

In this study, a liquid chromatography–tandem triple quadrupole mass spectrometer (AB SCIEX 5500, Framingham, MA, USA) was used, which was equipped with an electrospray ion source (ESI). The mobile phase for the column’s A phase consisted of a 0.05% aqueous ammonia solution, while phase B was acetonitrile. We injected a 5 μL sample at a column temperature of 35 °C and a flow rate of 0.3 mL/min. The gradient elution programme was as follows: phase B started at 10% for the initial minute (0–1 min), then increased to 50% over the next 0.5 min (1–1.5 min), and subsequently ramped up to 100% by 6 min, where it was maintained for 3 min. During the final stage, from 9 to 9.1 min, the proportion of phase B was reduced back to 10% and held for an additional minute.

The mass spectrometer was operated in negative ion mode with multi-stage reaction monitoring (MRM). The ion source temperature was set at 350 °C. The spray voltage was established at −4000 V, with the atomization temperature and nebulizing gas pressure set at 550 °C and 45 psi, respectively. The auxiliary gas pressure was maintained at 60 psi.

### 2.5. Method Validation

Each of the 20 samples were inserted to analyze one full procedure blank, one solvent blank and one matrix spiked sample; BPA and BPAF were detected in the full procedure blank, which were below the detection limit. The concentration range of the standard curve was 0.5 ng/mL–200 ng/mL, and the linearity of the standard curve was ≥0.995. The recoveries of the target spiked with the spiked concentration of 10 ng/mL were in the range of 68.4–112%, and the RSDs were in the range of 3.3–18%. To ensure accuracy and minimize potential drift, calibration standards were run at the beginning, middle and end of each analytical batch [18]. Furthermore, a quality control (QC) sample at a known concentration was analyzed every 10 samples to monitor instrument performance throughout the run. The measured values for the QC samples were consistently within the acceptable range (±15% of the nominal value).

### 2.6. Statistical Analysis

Descriptive statistics were used to describe demographic characteristics, food intake and levels of BPs in blood samples. Since the data collected in this study did not demonstrate normal distribution, the total score was thus expressed as the median M and interquartile spacing Q_U_-Q_L_, in which Q_U_ stands for the upper quartile (Q3) and Q_L_ stands for the lower quartile (Q1), and the rank sum test was used for between-group comparisons. Subsequently, correlations between BPs and food were tested by Spearman correlation analysis. Finally, because dietary levels were skewed, we therefore did not transform the BP data, but rather graded them by quartiles (Q1/Q2/Q3/Q4), and then assessed the effect of diet on the detection of BPs using ordered logistic regression analysis. All statistical methods were performed using SPSS 26.0, and *p* < 0.05 was statistically significant.

## 3. Results

### 3.1. Basic Information of Survey Population

A total of 161 elderly permanent residents in a community in Lianyungang were investigated, of which 104 were females, accounting for 64.6%; the average age of the respondents was 67.48 ± 5.06 years old, with the youngest being 57 years old and the oldest being 80 years old. The age distribution was as follows: 67 individuals were 65 years old or younger, while 94 were 65 years old and above. For body mass index (BMI), the normal population was 33.5%, the underweight population was 0.6%, the overweight population was 44.7% and the obese population was 21.1%. The study population had a normal diet, excluding those with a history of diabetes mellitus or intentional dieting. Most of the study population denied a history of smoking and alcohol consumption. Detailed information is shown in Table 1.

### 3.2. BP Internal Exposure in the Elderly

The BP levels in the serum of the investigated population were investigated, including BPA, BPB, BPC, BPE, BPF, BPAP, BPS, BPZ and BPP. It was found that BPA levels in the investigated population were significantly higher than the levels of other bisphenol compounds, with a range of 0.07–104.35 ng/mL and a median of 6 ng/mL.

**Figure 1:**
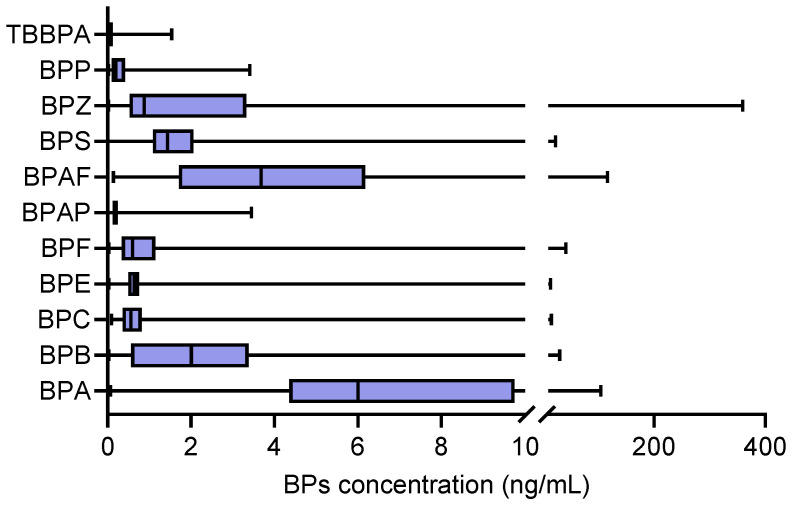
The levels of bisphenol analogues in the serum of elderly people in a community in Lianyungang, China (*n* = 161, ng/mL).

For other BPA substitutes, the median concentrations of BPB, BPAF and BPS reached 2.01, 3.68 and 1.44 ng/mL, with ranges of 0.14–116.25, 0–30.55 and 0–23.1 ng/mL, respectively (Table 2 and Figure 1).

### 3.3. Associations Between Serum BP Levels and Gender and Age

Having determined the internal levels of BPs, we then grouped the survey population according to gender and age. The total scores were expressed using the median M and interquartile spacing Q_U_–Q_L_, and the rank sum test was used for between-group comparisons. It showed that almost all types of BPs were higher in the male group than in females. In addition, almost all types of BPs were higher in the elderly population aged > 65 years than in those aged ≤ 65 years (Table 3).

**Figure 2:**
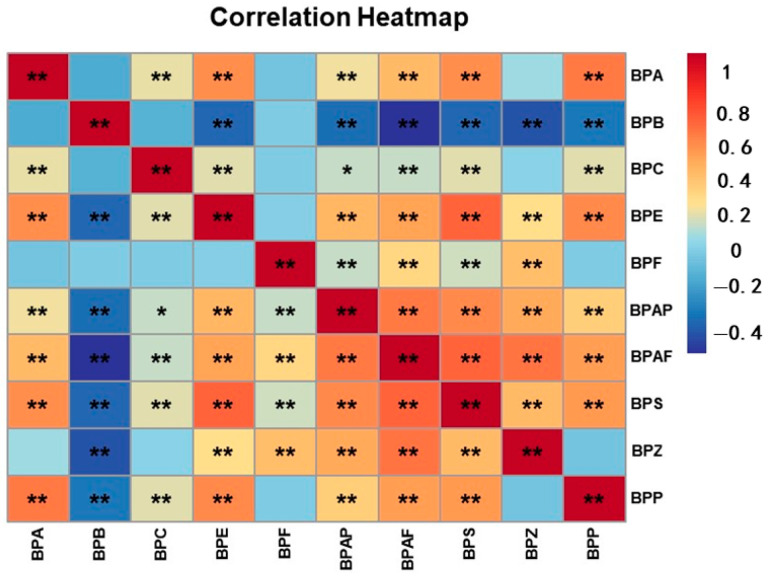
Heatmap of Spearman correlation analyses: associations among BPs. (** *p* < 0.01; * *p* < 0.05).

Considering that BPs are widely found in a variety of consumer products, including polycarbonate and other forms of plastics, food and beverage container linings, thermal printing paper and dental materials, people are therefore usually exposed to multiple BPs, and different BPs may have similar sources or routes of exposure. Also, BPS, BPF, BPB, etc., are inherently substitutes for BPA, so there are a variety of complex interactions. Therefore, we performed correlation analyses of different BPs, and these showed that there were significant correlations between almost all of the BPs. For example, there was a significant positive correlation between BPA and BPS (r = 0.571, *p* < 0.01) (Figure 2 and Table S2).

### 3.4. Relationship Between Serum BP Levels and Diet

**Figure 3:**
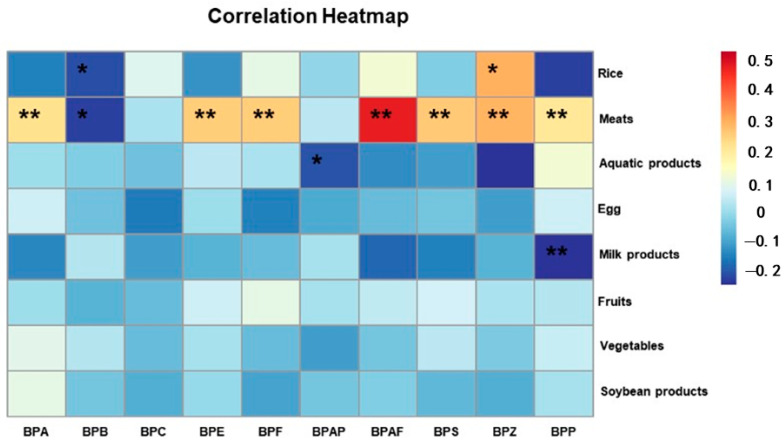
Heatmap of Spearman correlation analyses: associations among BPs and dietary situation. (** *p* < 0.01; * *p* < 0.05).

Diet is the main route of exposure to BPs, so we collected data on the diet of the abovementioned surveyed population, and a correlation analysis was performed between serum BPs and diet. Through the questionnaire survey, we found that the elderly population in the region mainly consumed staple foods such as rice. In addition to this, the intake of other foods, such as meat, vegetables and fruits, was balanced. The analysis found that almost all of the BPs showed a significant correlation with meat, except for BPC and BPAP, and the levels of BPA, BPE, BPF, BPAF, BPS, BPZ and BPP were all significantly and positively correlated with meat intake, which is presumed to be related to the lipophilicity of the BPs. In addition, a positive correlation was found between BPZ levels and rice intake. In addition, both BPAP and BPZ levels were negatively correlated with aquatic products (Table 4 and Figure 3). These results indicate that differences in lifestyle and dietary habits may contribute to regional differences in exposure to BPs.

## 4. Discussion

Our study revealed the serum BPS levels in older adults in coastal areas, and some similar studies have been conducted around the world. One report on an elderly population in Hubei, China, indicated that the median serum concentration of BPA was 3.09 ng/mL, with a range of 0.08–8.14 ng/mL [14]; and a study in a Malaysian population showed that the serum BPA levels ranged from 0.81 to 3.65 ng/mL [19]; while in a Thai population, the mean serum BPA level was 0.34 ng/mL, with a range of 0.071–0.746 ng/mL [20]. In Western countries, a study performed on a Swedish elderly population revealed that the median serum BPA concentration was 3.76 ng/mL, with a range of 2.02–6.52 ng/mL [21]. In a Chinese population study with 81 samples, it was found that the plasma concentrations of total BPs in adults ranged from 0 to 6.56 ng/mL, and the mean plasma concentrations (range) of BPA, BPS and BPAF were 0.40 (0–5.0) ng/mL, 0.15 (0–62.0) ng/mL and 0.073 (0–44.6) ng/mL, respectively. In serum samples collected from an elderly population living near e-waste recycling facilities in China, the concentrations of BPA, BPAF and BPF were 3.2, 0.0074 and 0.062 ng/mL, respectively [13], which were lower than those found in the coastal areas in this study. These results collectively indicate that BPA remains the prominent BP distributed globally. In the current study, slightly higher BPA levels, with a wider range of fluctuation, were observed, which may be presumed to be related to geographic location and population factors. Other studies are provided in Supplementary Table S2.

We found that serum BP levels were higher in most men than women, and higher in the elderly population aged >65 years than in those aged ≤65 years. In support of this, Wang et al. [13] reported that men had significantly higher urinary levels of BPA and BPS than women, which may be ascribed to smoking, alcohol consumption, dietary habits and high blood levels of androgens [19,22]. Research shows that serum elevated BPF and BPS exposure have negative associations with estrogen (E2) levels and E2/T (total testosterone) ratio, which may affect fertility [23]. In women with PCOS, serum BPS concentrations were found to be significantly higher compared to those in control subjects [7]. In 181 serum samples from pregnant Chinese women, ten BPs were positively identified and quantified by 0–144 ng/mL, of which TBBPS and BPS reached 0.593 and 0.113 ng/mL, respectively [24]. Some studies have found a gradual decrease in BPA concentrations with age, with significantly higher BPA levels in children than in adults, especially in those younger than 30 years of age [13,19,22,25], which may be associated with more frequent exposure to boxed lunches, beverages and other dietary habits. Free BPA is active and can be metabolized after ingestion, mainly by glucuronidation (BPA-G) and sulphation (BPA-S). The majority of BPA is metabolized in the liver through the production of BPA-G by the uridine 5′-bisphosphate glucuronide transferase (UDP-glycosyltransferase (UGT)) system, and is excreted by the kidneys [26]. In human hepatocytes, the contributions of glucuronidation and sulfation were found to be 94.04% and 5.96%, respectively [27]. Furthermore, it was shown that BPA had the highest intrinsic clearance, followed by BPF and then BPS [27]. With age, the activity of various enzymes in the body decreases, which we hypothesized to be one of the reasons for the higher levels of BPs in the elderly in the present study [28].

However, the concentration of BPs in residents near an electronics factory [29] was lower than that found in the coastal areas in this study; we speculated that this might be related to regional differences, so we investigated the eating habits of the study population, trying to determine the relationship between BPs and food.

The relationship between meat intake and higher serum BP levels may be due to the following reasons. Firstly, BPs in fresh meat may have been produced through environmental sources before slaughter. Animal feeds may contain BPs; Di et al. [30] selected feeds from different farms for detection, and found that most of the feed contained BPA, ranging from 1.0 ng/g to 50.0 ng/g. Meanwhile, samples from two farms were contaminated with BPA at concentrations ranging from 50.0 ng/g to 100.0 ng/g, and in one farm, BPA was found at levels even higher than 100.0 ng/g. In addition, BPA has been detected in feeds stored in woven bags, e.g., 0.52 μg/kg in cereals and 1.36 μg/kg in oilseeds, because the inner layer of a woven bag is polyethylene (PE) film [31]. BPA has also been detected in the feces or urine of livestock (e.g., cattle, pigs, sheep) and poultry (e.g., chickens, ducks and geese) [32]. Secondly, food conveyor belts, cutting boards, etc., are often used in processing and production, and these tools are usually made of PE, which is obviously contaminated with BPs [33]. Thirdly, different habits may also be one of the influencing factors of BP exposure. People who buy meat often directly use plastic bags, plastic boxes or cartons with plastic film, which are usually made of High-Density Polyethylene (HDPE) or Polypropylene (PP) [34], and there is a certain level of BP migration from the plastic bag to the meat [35]. In addition, more than 20 BPs, including BPA, BPS and BPAF, have been detected in takeaway containers made from Polystyrene (PS) plastic [36]. Finally, many households often use polycarbonate (PC) plastic containers to heat food, and after 9 min in a microwave oven, the average BPA concentration in hot pork cooked in PC containers increased to 14 ppb [37].

## 5. Conclusions

In this study, we selected 161 elderly permanent residents in an eastern coastal area of China as the research subjects, measured the concentrations of various BPs in their serum, and analyzed the differences in serum levels of various BPs among different genders and age groups. BPA levels were observed to be notably higher compared to the levels of other bisphenol compounds. Additionally, exposure levels of alternative compounds, such as BPB, BPAF and BPS, were also found to be elevated in human serum. It is important to note that BP concentrations tended to be generally higher in males as compared to females. Furthermore, examining the relationship between BP exposure and dietary habits uncovered a significant correlation between the consumption of meat products and the levels of most BPs. This finding offers novel insights and potential targets for interventions aimed at reducing BP exposure. Our dietary analysis revealed a significant correlation between meat consumption and serum BP levels, suggesting that meat is a major contributor to BP exposure in this population. Therefore, targeted interventions focused on reducing BP contamination in meat products could be effective in lowering overall BP exposure in elderly individuals. To the best of our knowledge, this is the first study of BP levels in a population-based serological sample in this region, filling a gap in the field.

## Figures and Tables

**Table 1:** Demographic characteristics of elderly people in a community in Lianyungang (*n* = 161).

Characteristics	*n*	Proportion (%)
Gender		
Male	57	35.4
Female	104	64.6
Age		
≤65	67	41.6
>65	94	58.4
Education level		
None	46	28.6
Elementary school (did not graduate)	9	5.6
Elementary school	24	14.9
Middle school	51	31.7
High school/middle school/technical school	28	17.4
Junior college	3	1.8
BMI		
Underweight	1	0.6
Normal weight	54	33.5
Overweight	72	44.7
Obese	34	21.1
High blood pressure		
Yes	60	39.5
No	101	60.5
Diabetes		
Yes	0	0.0
No	161	100.0
Smoking		
Never	120	74.5
Quit	12	7.5
Remain	27	16.8
Deficiencies	2	1.2
Drinking		
Regular	18	11.2
Sometimes	18	11.2
Never	123	76.4
Quit	2	1.2

**Table 2:** The levels of bisphenol analogues in the serum of elderly people in a community in Lianyungang, China (*n* = 161, ng/mL).

BPs	LOD	Detection Frequency (%)	Median	Min	P25	P75	Max
BPA	0.070	100	6.000	0.070	4.350	9.750	104.35
BPB	0.050	97.5	2.010	<LOD	0.570	3.380	30.55
BPC	0.200	78.9	0.560	<LOD	0.360	0.820	15.35
BPE	0.050	98.1	0.640	<LOD	0.500	0.750	13.8
BPF	0.060	99.3	0.600	<LOD	0.340	1.150	41.2
BPAP	0.007	96.4	0.160	<LOD	0.120	0.240	3.45
BPAF	0.003	100	3.680	0.140	1.710	6.170	116.25
BPS	0.010	98.8	1.440	<LOD	1.090	2.050	23.1
BPZ	0.040	96.3	0.880	<LOD	0.530	3.320	359.45
BPP	0.040	98.1	0.200	<LOD	0.100	0.410	3.41
TBBPA	0.100	24.2	< LOD	<LOD	<LOD	<LOD	1.54

LOD: limit of detection; SD: standard deviation; Min: minimum; Max: maximum; P25: percentile of 25; P75: percentile of 75.

**Table 3:** Analysis of differences in BP concentrations among different genders and age groups (M, QU–QL) (ng/mL).

BPs	Gender		Z-Value	*p*-Value	Age		Z-Value	*p*-Value
	Male (Median, Range)	Female (Median, Range)			≤65	>65		
BPA	6.43, 4.76–11.10	5.76, 4.08–8.83	−1.649	0.099	5.32, 4.06–7.28	7.08, 4.72–10.80	−2.445	0.014 *
BPB	2.13, 0.59–3.49	1.97, 0.56–3.36	−0.375	0.708	1.81, 0.53–3.10	2.24, 0.77–3.50	−1.320	0.187
BPC	0.57, 0.37–0.93	0.55, 0.33–0.74	−0.416	0.678	0.56, 0.41–0.73	0.56, 0.25–0.87	−0.190	0.850
BPE	0.63, 0.52–0.78	0.66, 0.50–0.75	−1.011	0.312	0.63, 0.50–0.72	0.67, 0.50–0.79	−1.087	0.277
BPF	0.64, 0.35–1.60	0.58, 0.34–1.09	−0.456	0.648	0.72, 0.40–1.31	0.56, 0.32–1.00	−1.595	0.111
BPAP	0.18, 0.12–0.25	0.15, 0.11–0.24	−0.969	0.333	0.16, 0.11–0.24	0.16, 0.12–0.24	−0.400	0.689
BPAF	3.75, 1.82–6.68	3.52, 1.64–5.95	−0.979	0.327	3.65, 1.70–6.94	3.70, 1.76–6.05	−0.322	0.747
BPS	1.43, 1.07–2.11	1.44, 1.09–2.02	−0.064	0.949	1.37, 1.12–1.98	0.50, 1.08–2.12	−0.333	0.739
BPZ	0.93, 0.57–7.95	0.89, 0.52–3.42	−0.636	0.525	0.88, 0.50–3.47	0.90, 0.61–3.87	−0.410	0.682
BPP	0.20, 0.11–0.46	0.20, 0.10–0.41	−0.937	0.349	0.21, 0.09–0.38	0.19, 0.11–0.44	−0.837	0.403

Note: * *p* < 0.05.

**Table 4:** Spearman correlation analyses of serum concentration of BPs and dietary situation.

	BPA	BPB	BPC	BPE	BPF	BPAP	BPAF	BPS	BPZ	BPP
Rice	−0.109	−0.167 *	0.094	−0.093	0.107	0.008	0.128	−0.003	0.289 **	−0.179
Meats	0.218 **	−0.179 *	0.035	0.251 **	0.248 **	0.053	0.452 **	0.254 **	0.285 **	0.204 **
Aquatic products	0.020	−0.006	−0.025	0.052	0.040	−0.164 *	−0.101	−0.075	−0.211 **	0.132
Egg	0.070	−0.024	−0.120	0.021	−0.111	−0.059	−0.036	−0.020	−0.077	0.075
Milk products	−0.107	0.042	−0.074	−0.045	−0.034	0.028	−0.139	−0.113	−0.047	−0.226 **
Fruits	0.022	−0.050	−0.032	0.073	0.108	0.029	0.061	0.078	0.035	0.041
Vegetables	0.100	0.047	−0.031	0.030	−0.031	−0.079	−0.021	0.053	−0.016	0.066
Soybean products	0.105	−0.023	−0.054	0.013	−0.068	−0.018	−0.006	−0.039	−0.056	0.033

* *p* < 0.05; ** *p* < 0.01 (2-tailed).

## Data Availability

The original contributions presented in the study are included in the article and Supplementary Materials; further inquiries can be directed to the corresponding authors.

## Supplementary Materials

The following supporting information can be downloaded at https://www.mdpi.com/article/10.3390/toxics13040259/s1: Table S1: Levels of BPA and other BPs in populations in different studies; Table S2: Results of Spearman correlation analyses: associations among BPs. Refs. [38,39] are cited in Supplementary Materials.
