# Supplementary material for: Internal Bisphenol Analogue Exposure in an Elderly Chinese Population: Knowledge from Dietary Exposure
Source: Toxics. 2025 Mar 29;13(4):259. doi: 10.3390/toxics13040259 (PMC12031501; doi:10.3390/toxics13040259)
Supplement: Supplementary file 1 [file toxics-13-00259-s001.zip › toxics-3519863-supplementary.pdf]

Supplementary Materials:

Table S1: Results of Spearman correlation analyses: associations among BPs.

	**BPA**	**BPB**	**BPC**	**BPE**	**BPF**	**BPAP**	**BPAF**	**BPS**	**BPZ**	**BPP**
BPA	1.000									
BPB	-0.048	1.000								
BPC	0.258**	-0.030	1.000							
BPE	0.577**	-0.253**	0.252**	1.000						
BPF	0.043	0.072	0.081	0.078	1.000					
BPAP	0.266**	-0.238**	0.196*	0.451**	0.205**	1.000				
BPAF	0.446**	-0.418**	0.204**	0.519**	0.348**	0.621**	1.000			
BPS	0.571**	-0.256**	0.239**	0.689**	0.207**	0.577**	0.681**	1.000		
BPZ	0.163*	-0.259**	0.156*	0.319**	0.459**	0.507**	0.626**	0.461**	1.000	
BPP	0.631**	-0.208**	0.246**	0.588**	0.072	0.364**	0.529**	0.544**	0.049	1.000

*p < 0.05; **p < 0.01 (2-tailed).

Table S2: Levels of BPA and other BPs in populations in different studies;

**Country**	**Detection method**	**Biological sample**	**Bisphenol analogues**	**Mean/Median (ng/ml)**	**Levels range (ng/ml)**	**ref**
Malaysia	GC/MS	human serum	BPA	-	0.81-3.65	[23]
Thailand	ELISA	human serum	BPA	0.34	0.071-0.746	[24]
Sweden	LC-MS/MS	elderly serum	BPA	3.76	2.02-6.52	[25]
Hubei	LC-MS/MS	elderly serum	BPA	3.09	0.08-8.14	[18]
Guangzhou	LC-MS/MS	human urine	BPA	31.07	10.21-106.77	[17]
China	LC-MS/MS	human plasma	BPA<br>BPS<br>BPAF	0.4<br>0.15<br>0.073	0-5.0<br>0-62.0<br>0-44.6	[29]

China	LC-MS/MS	human erythrocyte	BPA<br>BPS<br>BPAF<br>BPAP	0.21<br>0.035<br>0.017<br>0.013	0-0.7<br>0-0.18<br>0-0.11<br>0-0.38	[29]
U.S. and Asian countries	LC-MS/MS	Indoor dust	BPA<br>BPS<br>BPF	1.33<br>0.34<br>0.054	-<br>-<br>-	[42]
China	LC-MS/MS	human urine	BPA<br>BPS<br>BPF<br>BPAF	0.392<br>0.023<br>0.219<br>0.017	0-1.514<br>0-0.182<br>-<br>-	[43]
